# Supplementary material for: The causal relationship between the human gut microbiota and pyogenic arthritis: a Mendelian randomization study
Source: Front Cell Infect Microbiol. 2024 Nov 26;14:1452480. doi: 10.3389/fcimb.2024.1452480 (PMC11629706; doi:10.3389/fcimb.2024.1452480)
Supplement: Supplementary file 1 [file DataSheet1.zip › mendelian test/ebi-a-GCST90027695.csv_forest.pdf]

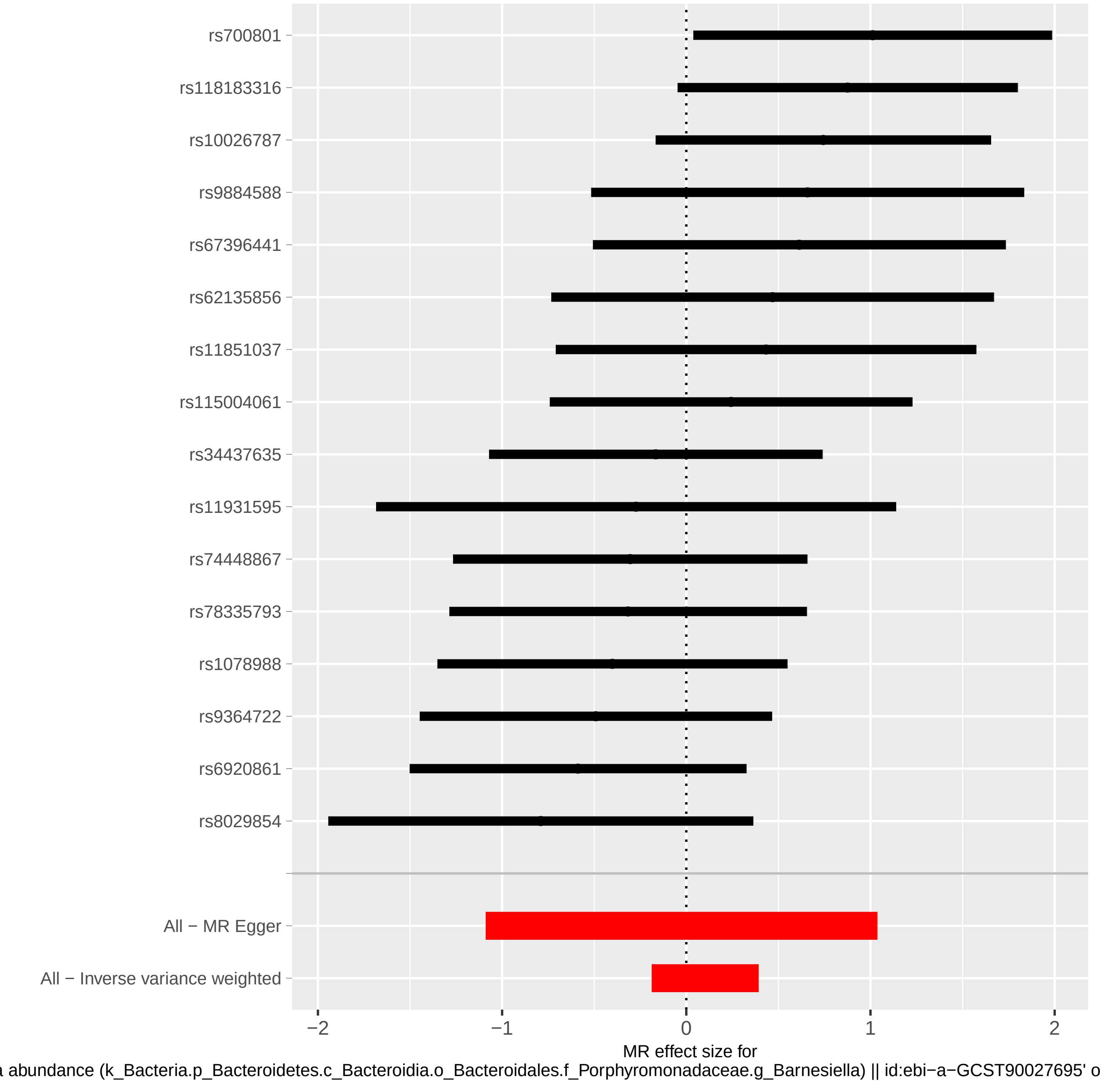
rs700801
rs118183316
rs10026787
rs9884588
rs67396441
rs62135856
rs11851037
rs115004061
rs34437635
rs11931595
rs74448867
rs78335793
rs1078988
rs9364722
rs6920861
rs8029854
All − MR Egger
All − Inverse variance weighted
−2
−1
0
1
2
MR effect size for
a abundance (k_Bacteria.p_Bacteroidetes.c_Bacteroidia.o_Bacteroidales.f_Porphyromonadaceae.g_Barnesiella) || id:ebi−a−GCST90027695' o
